# Transcriptomic Remodelling of Fetal Endothelial Cells During Establishment of Inflammatory Memory

**DOI:** 10.3389/fimmu.2021.757393

**Published:** 2021-11-19

**Authors:** Elisa Weiss, Amanda Vlahos, Bowon Kim, Sachintha Wijegunasekara, Dhanya Shanmuganathan, Thomas Aitken, Ji-Hoon E. Joo, Samira Imran, Rebecca Shepherd, Jeffrey M. Craig, Mark Green, Ursula Hiden, Boris Novakovic, Richard Saffery

**Affiliations:** ^1^ Perinatal Research Laboratory, Department of Obstetrics & Gynaecology, Medical University of Graz, Graz, Austria; ^2^ Molecular Immunity, Infection and Immunity Theme, Murdoch Children’s Research Institute, Parkville, VIC, Australia; ^3^ Department of Biosciences, University of Melbourne, Parkville, VIC, Australia; ^4^ Colorectal Oncogenomics Group, Department of Clinical Pathology, University of Melbourne, Melbourne, VIC, Australia; ^5^ University of Melbourne Centre for Cancer Research, University of Melbourne, Melbourne, VIC, Australia; ^6^ Department of Paediatrics, University of Melbourne, Royal Children’s Hospital, Parkville, VIC, Australia; ^7^ Molecular Epidemiology, Murdoch Children’s Research Institute, Parkville, VIC, Australia; ^8^ The Institute for Mental and Physical Health and Clinical Translation (IMPACT), School of Medicine, Deakin University, Geelong, VIC, Australia

**Keywords:** endothelial progenitor cell, endothelial cells, inflammation, trained immunity, inflammatory memory, innate immune memory, HUVEC (human umbilical vein endothelial cells), transcriptome (RNA-seq)

## Abstract

Inflammatory memory involves the molecular and cellular ‘reprogramming’ of innate immune cells following exogenous stimuli, leading to non-specific protection against subsequent pathogen exposure. This phenomenon has now also been described in non-hematopoietic cells, such as human fetal and adult endothelial cells. In this study we mapped the cell-specific DNA methylation profile and the transcriptomic remodelling during the establishment of inflammatory memory in two distinct fetal endothelial cell types – a progenitor cell (ECFC) and a differentiated cell (HUVEC) population. We show that both cell types have a core transcriptional response to an initial exposure to a viral-like ligand, Poly(I:C), characterised by interferon responsive genes. There was also an ECFC specific response, marked by the transcription factor ELF1, suggesting a non-canonical viral response pathway in progenitor endothelial cells. Next, we show that both ECFCs and HUVECs establish memory in response to an initial viral exposure, resulting in an altered subsequent response to lipopolysaccharide. While the capacity to train or tolerize the induction of specific sets of genes was similar between the two cell types, the progenitor ECFCs show a higher capacity to establish memory. Among tolerized cellular pathways are those involved in endothelial barrier establishment and leukocyte migration, both important for regulating systemic immune-endothelial cell interactions. These findings suggest that the capacity for inflammatory memory may be a common trait across different endothelial cell types but also indicate that the specific downstream targets may vary by developmental stage.

## Introduction

Immune memory is traditionally associated with adaptive immunity, mediated by specific antibody producing lymphocytes (1). This enables enhanced immune responses upon re-infection with a pathogen (2). In contrast to the adaptive system, innate immunity is traditionally described as the rapid, non-specific response to pathogens, involving the engagement of neutrophils, natural killer cells, monocytes, macrophages, the complement system and cytokines (1), in the absence of any capacity for memory. This dogma, ascribing memory solely to the adaptive immune compartment, has now been challenged, as an unequivocal body of literature has confirmed the memory capacity of specific cells within the innate immune system (3). A heightened response, relative to an initial stimulation, is often termed ‘trained immunity’ while a dampened or attenuated response is referred to as ‘tolerance’, phenomena that are on opposing ends of ‘inflammatory memory’ (4). Endotoxin tolerance is observed in sepsis patients (5), where an initial hyper-inflammatory response leads to a transient unresponsive state, usually characterised as inability to release proinflammatory cytokines (6). Trained immunity, on the other hand, is associated with stronger release of proinflammatory cytokines and has been associated with the off-target effects of the BCG vaccine (7) and is observed in monocytes in chronic conditions, such as obesity (8). Both phenomena are specified by epigenetic and metabolic remodelling upon initial microbial exposure, resulting in altered capacity for transcription of genes on subsequent challenge (9).

It has become clear that endothelial cells (ECs) play a key function in innate immune responses (10), becoming activated by inflammatory signals in association with increased capacity for cytokine production (11), cell permeability, leukocyte adhesion and transendothelial migration, as well as pro-coagulant features (12, 13). A growing body of evidence suggests that ECs are also capable of presenting antigens *via* MHC class II molecules (14). Thus, the immune system depends on the vascular response triggered by ECs (12). As with innate immune cells, recent data have revealed that ECs possess the capacity to establish inflammatory memory (15). Hence, modulation of endothelial immune response by immune memory effects may enable ECs to sense and respond more effectively, potentially to improve transendothelial guidance of immune cells to a site of inflammation (11). However, disruption of this process may also increase the susceptibility to develop endothelial dysfunction and chronic inflammation.

Initial exposure of murine adult ECs to pathogens increases leukocyte adherence following a subsequent challenge (16). Similarly, human adult aortic ECs exposed to oxidized low density lipoprotein (oxLDL) show a trained immune response following a second stimulation (15). In fetal ECs, such as HUVECs, an initial lipopolysaccharide (LPS) exposure leads to tolerance in response to a second LPS hit, while an exposure to the viral mimic Poly(I:C) induces a trained phenotype, and stronger cytokine (IL6 and CXCL10) response to a second LPS hit (17, 18). As ECs are highly heterogeneous in terms of the vascular bed from which they are derived (arterial *vs* venous, macro- *vs* microvascular), their developmental stage, and response to inflammatory stimuli, it remains unclear how generalisable the phenomenon of inflammatory memory is in ECs. We, and others, have also shown that endothelial cell heterogeneity is associated with epigenetic variation, including differences in DNA methylation profile (19, 20).

Although not widely studied in ECs it is clear that the capacity for training of innate immune cells also involves distinct epigenetic processes, such as active histone marks, open chromatin, and DNA methylation (21–24). Here, we hypothesised that the capacity for inflammatory memory is widespread in ECs of different tissue origin and developmental stage and is underpinned, at least in part, by common downstream molecular changes. In order to test this, we measured the capacity of fetal progenitor ECs (endothelial colony forming cells; ECFC) (25, 26) and more differentiated human umbilical vein ECs (HUVEC) (17, 18, 27) to establish inflammatory memory in response to a viral TLR3 ligand and characterised the transcriptional and DNA methylation profiles associated with this process in both cell types.

## Materials And Methods

### Isolation of HUVECs and ECFCs

HUVECs were extracted from umbilical cords, which were collected at delivery as part of the Peri/Postnatal Epigenetic Twins Study (PETS) (28). Briefly, type 2 collagenase (1 mg/mL, Worthington Biochemical Corporation, Lakewood, NJ, USA) was used to detach endothelial cells which were then further purified using CD31 MicroBead Kit (Miltenyi Biotec, Bergisch Gladbach, Germany) according to the manufacturer’s instructions. Explant cultures were established and viably frozen in Fetal Calf Serum (FCS) with 10% DMSO at passage 2-4. Fetal ECFC isolation was approved by the ethics committee of the Medical University of Graz, Austria, (29-319 ex 16/17) and written informed consent was obtained. ECFCs were isolated from umbilical cord blood *via* density gradient centrifugation and characterized as described (29). Samples were frozen in culture media with 20% FCS and 10% DMSO at passage 3. All primary lines used in this study are from healthy term pregnancies of women in normal weight range without any pregnancy complications. Exclusion criteria were overweight/obesity, diabetes, hypercholesterolemia, and acute/chronic diseases. Placental and fetal weight and height are in normal range, revealing no sign of any inflammatory disease.

### Inflammatory Memory Model

Endothelial cells were passaged at 70-90% confluency and seeded in 6-well plates coated with 1% gelatine at a density of 1.5-2.5 x10^5^ (Nunclon Delta, Thermofisher). Cells were then left to attach for 24h at 37°C, 21% O_2_, 5% CO_2_ in EGM-2 media (Lonza, Basel, Switzerland). Following attachment, cells were stimulated with Poly(I:C) (10 µg/mL, Sigma, Burlington, MA, USA) or media only for 24h. Poly(I:C) was removed after 24h, replaced with EGM-2 only, and cells were left to rest for a further 24h. Cells were then re-stimulated with 100 ng/mL LPS (Sigma) for 4h (**Figure 2A**). Endothelial cells in 6-well culture plates were collected at T0, 4h, 24h, 48h, and 52h in 500 µL RLT+β-mercaptoethanol (Qiagen, Venlo, Netherlands). All experiments were performed in duplicate, using cells between passages 4-6.

### RNA Collection and Sequencing

Total RNA was extracted from cells using the RNeasy RNA extraction kit (Qiagen) with on-column DNaseI treatment. RNA quality (RIN) scores were determined using the RNA Tapestation system (Agilent). Libraries were prepared by the Victorian Clinical Genetic Services (VCGS) Sequencing Service (Melbourne, Australia) using the TruSeq stranded mRNA kit (Illumina). Libraries were sequenced on the NovaSeq 6000 (Illumina) at ~20 M reads per sample, using 2x150 bp reads.

### cDNA Synthesis and Quantitative Reverse Transcription PCR

cDNA was synthesised using the Tetro cDNA Synthesis Kit (Meridian Bioiscience) as per the manufacturer’s protocol. Gene expression levels were analysed by quantitative real-time PCR using LightCycler 480 (Lifesciences, Roche). cDNA was amplified using primers ordered from Integrated DNA Technologies (IDT), reconstituted to 100 µM as per the manufacturer’s instructions, then diluted to a 10 µM working solution (20µL Forward primer, 20 µL Reverse primer, 160 µL Nuclease free H2O) for IL-6 ([F-AAAGAGGCACTGGCAGAAAA] [R-AGCTCTGGCTTGTTCCTCAC]), MX1 [F-GTGCATTGCAGAAGGTCAGA] [R-GGATGATCAAAGGGATGTGG], CCL2 [CCCCAGTCACCTGCTGTTAT] (F) [TGGAATCCTGAACCCACTTC] (R), and GAPDH ([F-TTCGACAGTCAGCCGCATCTT] [R-CCCAATACGACCAAATCCGTT]). qPCR mixtures were prepared in 10 µL reactions comprised of 5 µL of SensiFast SYBR Green I mix (BIO-98050) (Meridian Bioscience/BIOLINE), 1 µL of combined forward and reverse primers (1 µM final concentration), 2 µL of nuclease-free H_2_O, and 2 µL of diluted cDNA template (diluted 1:10 with nuclease-free H_2_O). Samples and no template controls (NTCs) were amplified in triplicate, and HUVEC and ECFC expression levels were determined using the ΔΔCt method, with *GAPDH* as the control gene.

### RNA Sequencing Analysis

To infer gene expression levels, RNA-seq reads were aligned to hg19 human transcriptome using Bowtie (30). Quantification of gene expression was performed using MMSEQ (31). Counts per gene were normalised using DESeq2, while RPKM values were used for plotting gene expression in heatmaps and line graphs. DESeq2 was used to identify differentially expressed genes (DEGs) using logistical regression (32), with p value <0.05, fold change >2 and RPKM >1 considered significant. Pairwise comparisons were performed over time (time 0, 4 hours, 24 hours, 48 hours and 52 hours) and between stimulations [media *vs* Poly(I:C)]. The gene lists were then merged, and duplicates removed, to understand the time-course dynamics using PCA plots using prcomp in R. For 4h LPS exposure experiments, DEGs were identified as p value <0.05, fold change >2 and RPKM >1. Trained, unaffected and attenuated genes were identified by comparing media+LPS to Poly(I:C)+LPS, with fold change >1.5 higher in Poly(I:C)+LPS designated as trained, fold change >1.5 higher in media+LPS as attenuated, and all other genes assigned as unaffected. Gene ontology and promoter motif enrichment was performed using HOMER (33).

### DNA Methylation Analysis

Raw Infinium HumanMethylation.idat files (Illumina, San Diego, CA) were downloaded from the Gene Expression Omnibus (GEO) for healthy HUVECs (GSE103253), ECFCs (GSE180355), placental arterial endothelial cells (AEC) and venous endothelial cells (VEC) (GSE106099) (34, 35). Raw.idat files were processed and analyzed using the MissMethyl and minfi packages for R (36, 37). Samples were checked for quality and those with a mean detection p-value of >0.01 were removed. Data were normalized for both within and between array technical variation using SWAN (Subset-quantile Within Array Normalization) (38). Probes with poor average quality scores (detection p-value > 0.01), those associated with SNPs (MAF > 0%) and cross-reactive probes (39) were removed from further analysis. Differential methylation analysis by linear regression modelling was performed using limma (40). DMPs were assigned to the nearest gene within 1Mb using the GREAT tool (41).

### Data Availability Statement

The data sets generated and analyzed for the current study are deposited in the Gene Expression Omnibus repository with the accession number GSE180881.

## Results

### Fetal Endothelial Cells of Different Origins Display Distinct Immune-Related Genome-Wide DNA Methylation Patterns

**Figure 1:**
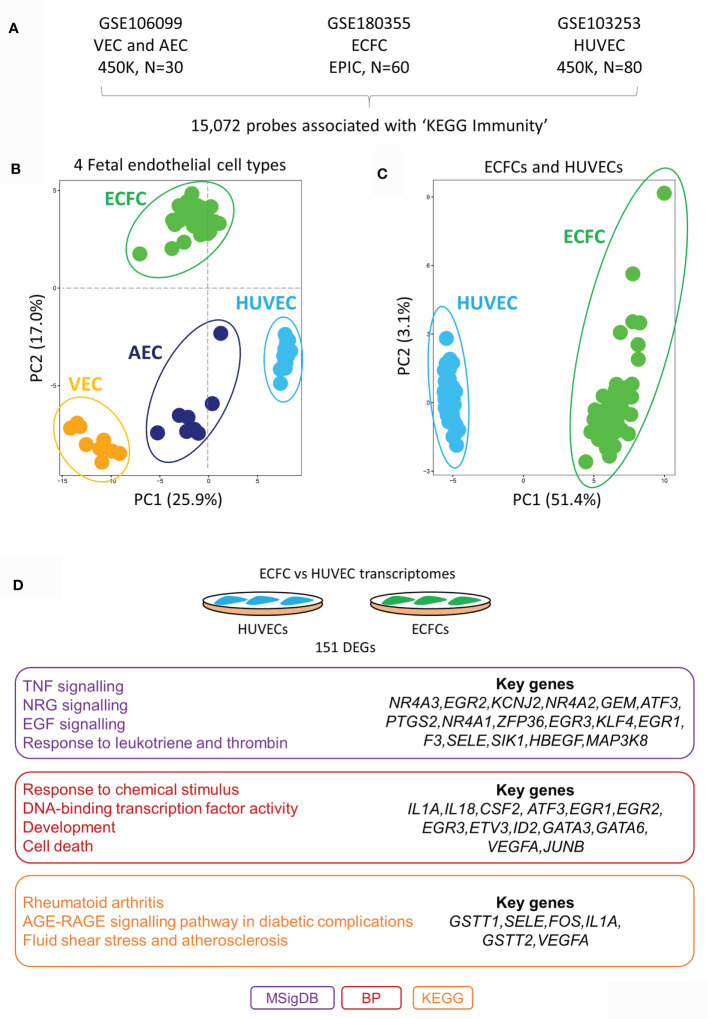
DNA methylation and transcriptomic profiles of immunity-related genes in different placental endothelial sub-types. **(A)** Immunity-related probes were extracted from publicly available Infinium HumaMethylation datasets for VEC (450K platform), AEC (450K platform), ECFC (EPIC platform) and HUVECs (450K platform). **(B)** PCA plot of immune-related probes at rest. HUVECs are separated *via* PC1 and ECFCs *via* PC2 from the other fetal endothelial cell types. **(C)** PCA plot of immune-related genes at rest in ECFCs and HUVECs. **(D)** Differentially expressed genes between ECFCs and HUVECs at baseline. Gene ontology terms associated with the differentially expressed genes. MSigDB, GSEA Molecular Signatures Database; BP, Gene Ontology Biological Process; KEGG, Kyoto Encyclopedia of Genes and Genomes.

To test our hypothesis, we first mapped the epigenetic and transcriptional landscape of immune-related genes in a range of ECs at rest (unstimulated; **Figure 1**). Using published genome-wide methylation data from HUVECs (n=80), cord blood-derived ECFCs (n=60), placental arterial ECs (AECs) (n=15) and placental venous ECs (VECs) (n=15), we extracted methylation data for 15,072 probes within 100kb of genes related to the KEGG section ‘5.1 - Immune system’ (**Figure 1A**). These probes were chosen because we wanted to specifically focus on DNA methylation differences that may influence inflammatory gene expression responses in cis. Principal component analysis (PCA) clustering according to methylation data of these probes clearly separated the four cell types, HUVECs from the rest (PC1) and ECFCs from the rest (PC2) (**Figure 1B**). Clustering of ECFCs and HUVECs alone shows a strong signature (**Figure 1C**), which we postulated will result in differing responses to microbial ligands and capacity for inflammatory memory. Next, we compared the baseline transcriptomes of HUVECs and ECFCs, revealing a total of 151 genes with differential expression (DEGs) (**Figure 1D** and **Table S1**). Amongst the differentially expressed genes were those involved in inflammatory response, EGF signalling, transcriptional regulation, and response to environment (**Figure 1D**). These epigenetic and transcriptional signatures suggest that the two cell types may mount a distinct transcriptional inflammatory response.

### Poly(I:C) Induces Large-Scale Transcriptional Remodeling in Fetal Endothelial Cells

**Figure 2:**
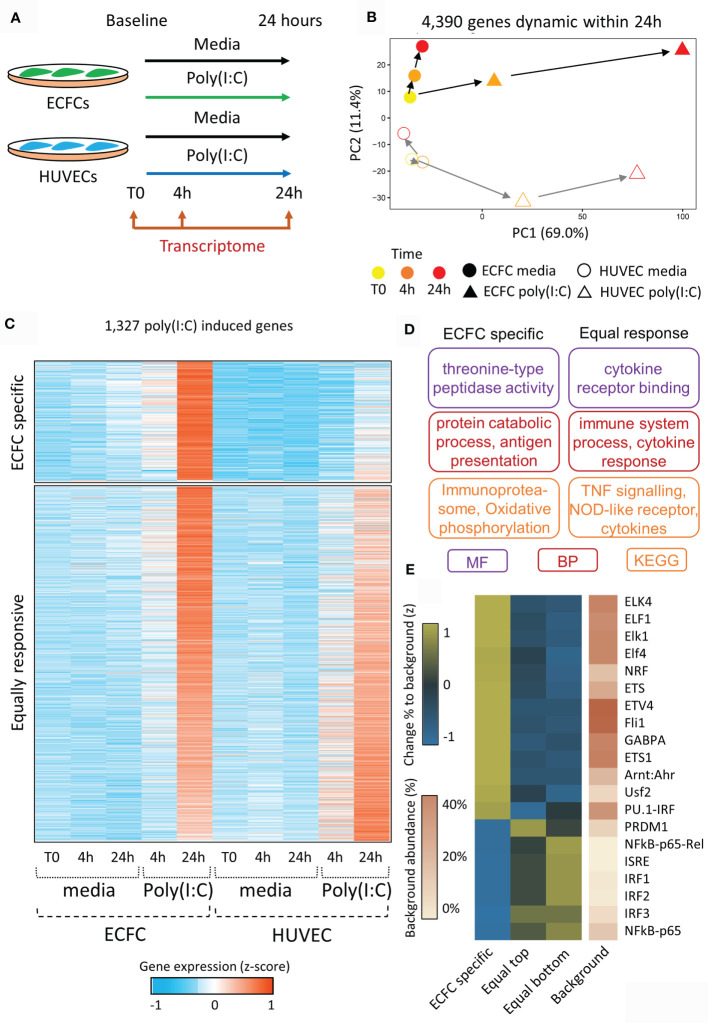
Transcriptional reprogramming of ECFCs and HUVECs in response to viral mimic Poly(I:C). **(A)** Experimental model. **(B)** PCA plot of transcriptomic changes that occur 4 hours and 24 hours following Poly(I:C) exposure. PC1 shows the response to Poly(I:C), which peaks at 24h, while PC2 represents the difference between ECFCs and HUVECs. **(C)** Heatmap showing genes upregulated by Poly(I:C) at 24h. A subset of genes is only induced in ECFCs, while the majority are equally induced in both cell types. **(D)** Gene ontology terms associated with ECFC-specific and equally induced genes. **(E)** Heatmap showing enrichment of transcription factor motifs at promoters of ECFC-specific and equally induced genes.

The viral dsRNA mimic, Poly(I:C), was chosen as the acute stimulus due to its ability to induce inflammatory memory in HUVECs (17, 18). Each of ECFCs (n=2) and HUVECs (n=2) were exposed to Poly(I:C) in culture for 24 hours with RNA isolation and sequencing (RNA-seq) at baseline (T0), 4 hours (4h), and 24 hours (24h) (**Figure 2A**). We performed pairwise comparisons across time for each exposure [e.g. ECFC 4h Poly(I:C) *vs* ECFC 24h Poly(I:C)], and between exposures at matched time-points (e.g. HUVEC 4h media *vs* HUVEC 4h Poly(I:C)). A total of 4,390 protein-coding genes were differentially expressed in our model across all comparisons (FC >2, p <0.05), which we visualised using a PCA plot, with the Poly(I:C) response being highest at 24h post exposure (PC1; **Figure 2B**). This Poly(I:C) response trajectory was similar between the two cell types, even if the cell-type specific transcriptional profiles persisted (PC2; **Figure 2B**).

In order to determine the trajectory of Poly(I:C) induced genes following the removal of the stimulus, we profiled the 4,390 dynamic genes at the 48h time-point, 24h after the removal of Poly(I:C) (**Figure S1**). We separated Poly(I:C) induced genes into 4 quartiles (Q1 to Q4) based on their expression at the 48h time-point. Q1 genes showed the most persistent elevated expression at 48 hours, while Q4 genes returned to basal levels (**Figure S1A**). We confirmed that this was a direct result of Poly(I:C) exposure, and not simply a persistence of low levels of Poly(I:C) remaining after media replacement (**Figure S2**). Promoters of genes showing persistent expression were enriched for the interferon stimulated response element (ISRE) and viral response pathways. The viral response gene, *MX1*, was the top gene in the persistent expression (Q1) quartile in both ECFCs and HUVECs, while *TNF* expression returned to baseline by 48h (Q4) (**Figure S1B**). There was no clear promoter-associated TF signature in genes that returned to baseline, with no motif reaching a p-value or fold change threshold relative to background (**Figure S1C**).

### ECFCs and HUVECs Show Distinct Transcriptional Responses to Poly(I:C)

In total, 1,327 genes were specifically upregulated following 24h of Poly(I:C) exposure in either ECFCs or HUVECs (**Figure 2C** and **Table S2**). Of these, 1,039 were induced in both cell types (‘equally responsive’), indicating that the two endothelial cell types share common inflammatory pathways (**Figure 2C**). These genes are involved in cytokine receptor signalling and TNF signalling pathways (**Figure 2D**) and their promoters were enriched for viral transcription factor motifs, such as the ISRE, IRF1-3, and NFKB (**Figure 2E**). This motif signature was particularly strong at genes that were slightly more responsive in HUVECs (bottom half of heatmap, **Figure 2C**). Expression of genes coding for transcription factors associated with the motif signature was higher in HUVECs than ECFCs (**Figure S3**). There was also an ECFC-specific gene signature, containing 288 genes that were non-responsive in HUVECs at 24h Poly(I:C) exposure (**Figure 2C**). This set of genes was enriched for ‘oxidative phosphorylation’, ‘immunoproteasome’ and ‘antigen presentation’ (**Figure 2D**), and their promoters were marked by motifs recognised by ELF1 (**Figure 2E**). Due to the fundamental role of metabolic remodelling in endotoxin tolerance and trained immunity in macrophages (42, 43), we specifically looked at genes in the ‘glycolysis’, ‘oxidative phosphorylation’ and ‘metabolism’ GO categories (**Table S3**). In total, 4,390 (21.9%) protein coding genes were dynamically expressed in ECFCs or HUVECs during the 48-hour experimental set-up in response to media or Poly(I:C). Metabolic genes overall were not more dynamic (22.1%, 380/1,714 genes), however 29.9% (32/107) of genes involved in oxidative phosphorylation were dynamic (**Table S3**). The same increase was observed for genes responsive to LPS re-stimulation, with 3.4% of all protein coding genes dynamic, while 10.3% of oxidative phosphorylation genes were altered.

### Specific Poly(I:C) Responses Are Associated With Minimal DNA Methylation Variation

**Figure 3:**
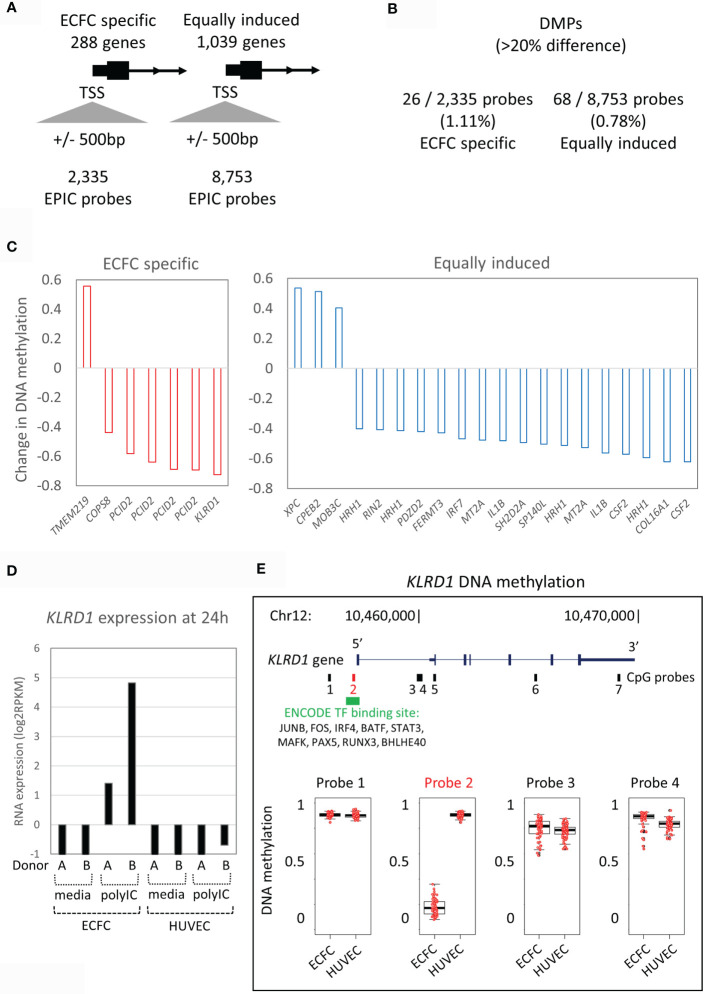
DNA methylation patterns at promoters of Poly(I:C) induced genes. **(A)** The 288 ECFC-specific Poly(I:C) induced genes have 2,335 EPIC probes at their promoters, while the 1,039 equally induced genes have 8,753 EPIC probes. **(B)** Only 1.1% and 0.8% of probes are differentially methylated (DMPs) between ECFCs and HUVECs. **(C)** Bar plot showing mean difference in DNA methylation between ECFCs and HUVECs at top promoter DMPs. **(D)** Bar plot showing expression of *KLRD1* in ECFCs and HUVECs after 24h exposure to media or Poly(I:C). **(E)** Map of the KLRD1 locus, showing EPIC probes and TF binding tracks. Of the 7 EPIC probes, only one is differentially methylated between ECFCs and HUVECs. Probe 2 is present at the transcriptional start site (TSS), overlaps TF binding sites and shows complete hypomethylation in ECFCs, predicting responsiveness.

Next, we tested our hypothesis that the expression difference to the primary Poly(I:C) response between ECFCs and HUVECs (**Figure 2C**) is associated with the underlying DNA methylation differences between the two EC types (**Figure 3**). We restricted this analysis to promoter differential methylated probes (DMPs) to ensure gene-specificity. To do this, we extracted all CpG probes from the gene promoter regions that mapped to within 500bp of the transcriptional start site (TSS) that showed ‘ECFC-specific’ or ‘equally responsive’ patterns (**Figure 3A**). Only 1.1% (26/2,335) of probes associated with ‘ECFC-specific’ genes, and 0.8% (68/8,753) of probes mapping to ‘equally responsive’ genes showed differential methylation between the two cell types at a stringent 20% absolute cut-off (**Figure 3B**). In each of these rare instances, only a single DMP was present at the promoter. This indicates that the underlying DNA methylation profile within the promoter regions is a poor predictor of response to Poly(I:C). Nevertheless, the top ranked ECFC-specific gene, *KLRD1* (**Figure 2C**), had a single DMP in its promoter region (**Figure 3C**). This gene is inducible by Poly(I:C) in ECFCs only and is not expressed in HUVECs at any time, or in response to stimulation (**Figure 3D**). The DMP is in the promoter region, showing complete hypomethylation in ECFCs and overlapping an ENCODE TF binding site (**Figure 3E**).

### Evidence of Inflammatory Memory in HUVECs and ECFCs

**Figure 4:**
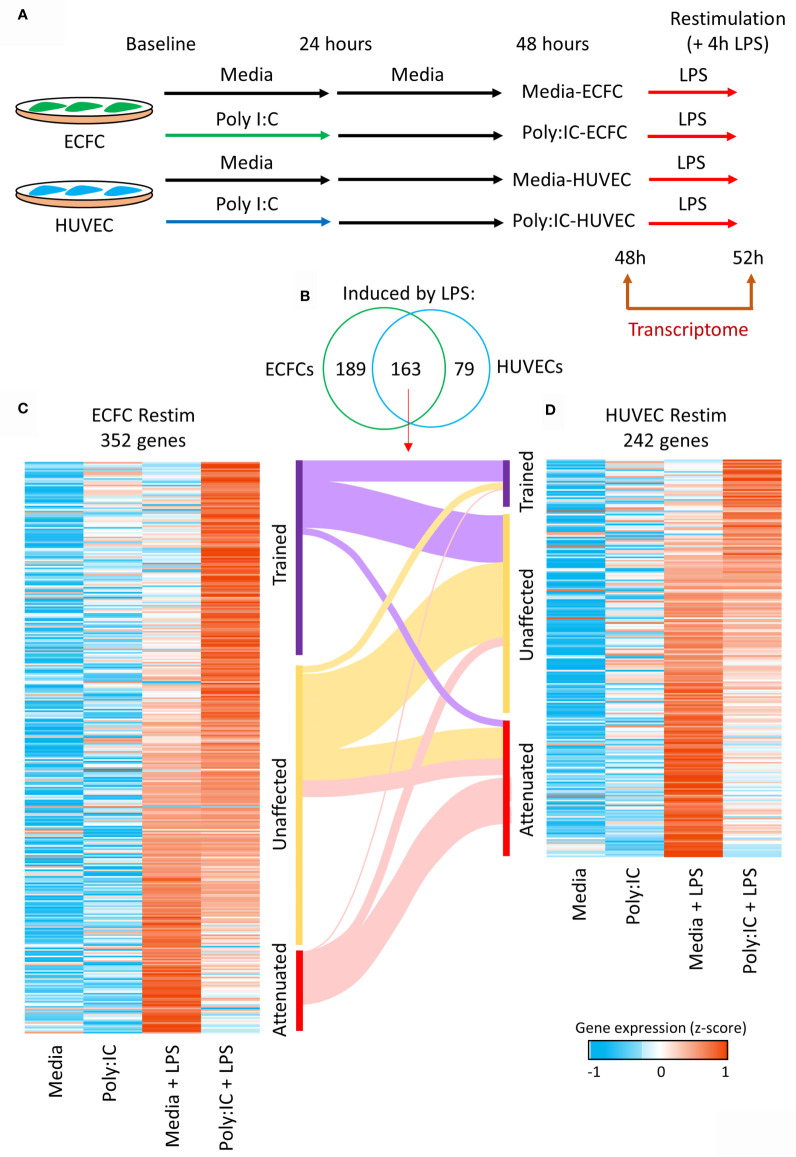
Trained immunity in ECFCs and HUVECs. **(A)** *Ex vivo* model to test Poly(I:C) induced trained immunity in fetal endothelial cells. Cells were exposed to Poly(I:C) for 24h, followed by 24h rest, and re-stimulation with LPS, an unrelated microbial compound. RNA-seq data was generated at 48h and 52h (4h after LPS exposure). **(B)** A total of 352 and 242 genes are induced by LPS in ECFCs and HUVECs, respectively, of which 163 are induced in both. **(C, D)** Heatmap of the LPS inducible genes in ECFCs and HUVECs, ranked from trained to tolerized by Poly(I:C). A Sankey plot shows the overlap between trained, equal and tolerized genes in ECFCs and HUVECs.

Next, we explored the influence of Poly(I:C) primary exposure on a secondary unrelated bacterial, lipopolysaccharide (LPS), transcriptional response (**Figure 4A**). After an initial 24h Poly(I:C) exposure, cells were allowed to rest for another 24h in media alone, after which they were stimulated for 4h with LPS (17, 18). A total of 352 genes showed expression changes induced by LPS in media-ECFCs or Poly(I:C)-ECFCs (**Figures 4B, C** and **Table S4**), and 242 genes were induced by LPS in media-HUVECs or Poly(I:C)-HUVECs (**Figures 4B** **, D** and **Table S5**). Each EC type displayed examples of both trained (heightened induction) and tolerized (reduced induction) gene expression in response to LPS, with HUVECs having more tolerized genes **(** **Figure 4D** and **Figure S4A**), and ECFCs more trained genes (**Figure 4C**). A total of 163 common genes were induced in both cell types (**Figure 4B**), with a general concordance in the direction of effect on gene expression (**Figures 4C, D**).

**Figure 5:**
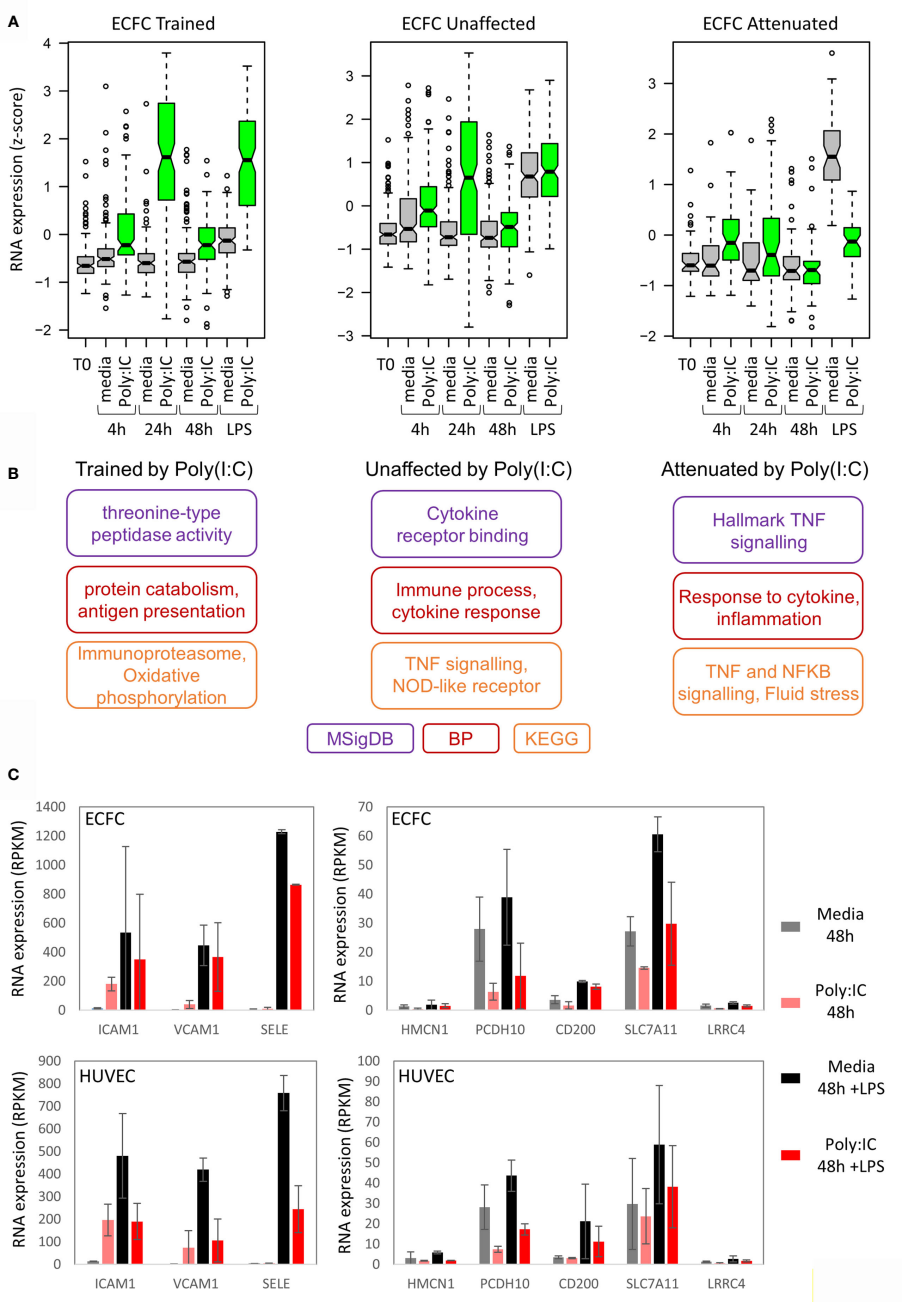
Poly(I:C) tolerizes genes involved in endothelial cell adhesion. **(A)** Expression of trained, unaffected and tolerized genes over time in ECFCs. This indicates that genes trained for LPS response are more strongly induced by Poly(I:C) initially compared to genes that are tolerized for LPS response. **(B)** Gene ontology analysis for each group of genes. **(C)** Expression of cell-adhesion genes that are tolerized for LPS response by Poly(I:C).

In general, the level of induction by the initial Poly(I:C) response determined the LPS response. For example, genes that were trained for LPS response by Poly(I:C) were more strongly induced by Poly(I:C) than those that were tolerized (**Figure 5A** and **Figure S4B**). To understand the underlying pathways involved in inflammatory memory in endothelial cells, we performed gene ontology analysis on genes that showed trained, unaffected or tolerized responses to re-stimulation with LPS (**Figure 5B**). Genes involved in inflammation were enriched in all three groups. Interestingly, the attenuated gene set was enriched for leukocyte transendothelial migration, with *SELE*, *ICAM1*, and *VCAM1* genes in the list (**Figure 5C**).

## Discussion

Innate immune memory is a relatively novel phenomenon with implications for a range of human inflammatory disorders, infectious disease, and vaccinology (4, 9). The establishment of innate immune memory involves metabolic, epigenetic and transcriptional remodelling, which subsequently influences responses to secondary stimuli (23, 42, 44). This phenomenon is not restricted to cells of the hematopoietic lineage, indicating that memory to microbial compounds, danger signals or injury can be established in a range of cell types (45).

In this study we selected two human fetal endothelial cell types, HUVECs and umbilical cord blood ECFCs, which derive from circulating endothelial progenitor cells, to investigate transcriptome remodelling during development of inflammatory memory. Two biological replicates was based on our previous work on monocyte inflammatory memory (23) and allows us to only detect robust changes in gene expression. However, we did not have enough statistical power to contrast the memory signature in ECFCs to that in HUVECs (**Figure 5**). ECFC are recruited for repair, vascular growth and angiogenesis (46) and their high abundance perinatally (26) suggests a function in postnatal vasculogenesis and angiogenesis. Our data shows that both cell types can mount a transcriptional response to Poly(I:C) exposure (**Figure 2**), which influences the transcriptional output to a second stimulation with LPS (**Figure 4**). Similarly to trained macrophages, genes involved in oxidative phosphorylation were more likely to be transcriptionally dynamic in response to Poly(I:C) or LPS in endothelial cells (**Table S3**). This indicates that metabolic remodelling is a common event in the establishment of inflammatory memory in different cell types.

Due to inexperienced adaptive immunity, innate immunity is critical for neonatal survival. The fact that stimulation of TLR3 with agonists induces inflammatory gene expression and interferon production in human adult and fetal endothelial cells highlights a common role of fetal and adult endothelial cells in innate immunity (17). Our study specifically identified upregulation of genes encoding interferon-inducible proteins (e.g. *MX1*, *MX2*) and interferon inducible transmembrane protein (e.g. *IFITM1*, *IFITM2*) which are known anti-viral genes. Particularly, interferon-inducible transmembrane (IFITM) proteins inhibit viral entry, transcriptional processes and protein synthesis (47). This was shown in both HUVECs (fetal ECs) and adult endothelial cells (48). The TLR3-mediated expression of antiviral genes in HUVEC and ECFC suggests that neonatal endothelial cells contribute to the vital/essential innate immunity of the newborn.

The participation of ECs in immune response is linked to the development of endothelial dysfunction and cardiovascular disease (CVD). For instance, anti-inflammatory cytokines can inhibit endothelial dysfunction (49) whilst endothelial dysfunction represents a shift towards a pro-inflammatory state, inducing CVD (50). Early development is particularly sensitive towards programming events. In fact, environmental influences and maternal pathologies in pregnancy affect long-term health and disease of the offspring in later life (51). Moreover, inflammatory situations such as infections in early life could increase the risk of CVD in adulthood (52). The fact that the immune response of fetal ECs can be programmed by exposure to pyrogens suggests that endothelial inflammatory memory *in utero* may participate in this programming event.

Environmental influences of various kinds alter the epigenome of cells, leading to memory effects and programming. In fact, primary ECs are modified by, for instance, inflammatory environment and hyperglycemia *in vitro* (53) and *in vivo* (34). Moreover, programming effects have not only been observed in mature ECs, but also in cord blood ECFCs (54). Thus, when using primary cells in the field of cell engineering, epigenetic and transcriptional adaptations to the donors’ environment are to be expected. These adaptations cannot be avoided but can probably be minimized by using cells from donors that do not suffer from active infections as well as chronic diseases. The advantage of using cord blood ECFCs for cell therapy is also highlighted by a study that showed a reduced pro-inflammatory signature in cord blood ECFCs compared to adult ECFCs (55). In addition to transcriptomics, future studies should explore the differences between cord blood and adult ECFCs at the level of epigenetics and metabolomics, as all of these are known to influence inflammation and trained immunity in general (42).

In support of the theory that the heterogeneous functions of ECs are specified by underlying molecular differences, we confirmed that ECFCs and HUVECs represent two epigenetically distinct fetal endothelial cell populations (**Figure 1**). Further, the differential response of these cells to stimuli [Poly(I:C)] was also characterised by differential gene expression patterns (**Figure 2**). Interestingly, Poly(I:C) induced the expression of 288 genes exclusively in ECFCs (**Figure 2**), which were related ‘oxidative phosphorylation’, ‘immunoproteosome’ and ‘antigen presentation’. Threonine-type peptidases are central components of the immunoproteasome. It is still controversial whether ECs are capable of antigen presentation. However, the fact that in microvasculature and small vessels, in addition to MHC class I antigens, ECs express MHC class II antigens *in vivo* points to this ability (56, 57). *In vitro*, endothelial MHC expression diminishes and requires cytokine stimulation to recover, and cultured ECs pretreated with interferon gamma (IFN-γ) to express MHC II, activate CD4+ central memory (T_CM_) and effector memory (T_EM_) T-cells (58). This ECFC-specific signature was marked by enrichment for ELF promoter motifs, as opposed to the IRF signature in the equally induced genes (**Figure 2E**). ELF1 is an ETS transcription factor that regulates a viral response that is distinct from interferon (59), suggesting that this pathway is active in ECFCs, but not HUVECs.

Only a small proportion of observed transcriptional responses in ECFC *vs* HUVEC could be explained by differences in baseline DNA methylation profile, suggesting that other molecular mechanisms, such as histone modifications, may underlie their distinct immunological phenotype and function. Histone modification have a shorter half-life than DNA methylation and are more responsive to acute stimuli (44). In particular histone 3 lysine 4 tri-methylation (H3K4me3) is enriched at promoters of pro-inflammatory cytokines in trained monocytes (43). Therefore, future studies should explore genome-wide histone modifications to identify regulatory elements that control inflammatory transcriptional responses in fetal ECs. Nor can the differences be explained by differences in TLR3 and TLR4 expression levels, which are expressed at similar levels in the two cell types. Interestingly, one CpG site in the promoter for *KLRD1* (killer cell lectin like receptor D1) was completely hypomethylated in ECFC, enabling the induction of *KLRD1* gene expression, whilst *KLRD1* transcripts were virtually absent in HUVECs before or after Poly(I:C) exposure. Interestingly, *KLRD1* (CD94) is reported to encode an NK cell-specific receptor, though some evidence of non-NK expression also exists, including ECs (Human protein atlas). Binding of HLA-E to KLRD1 prevents the cytotoxic activity of NK cells (60). The function of KLRD1 in ECFC however, remains topic of further studies.

Inflammatory memory in HUVECs has recently been reported at the level of cytokine release (17, 18). Our data demonstrates that both ECFCs and HUVECs can establish transcriptional inflammatory memory, with some differences in terms of degree of memory and the number of genes that are trained or tolerized (**Figure 4**). In general, there was concordance in direction with genes trained in one cell type likely to be trained in the other (**Figures 4C** **, D**). ECFCs induced a higher number of genes after LPS re-exposure, which is in line with these cells being microvascular ECs (61). For instance, lung microvascular endothelial cell produce larger amounts of cytokines upon stimulation (62) and L-selectin dependent adhesion of leukocytes occurring on microvascular but not macrovascular ECs of the human coronary system (63).

An interesting pathway that was tolerized in both ECFCs and HUVECs was leukocyte transendothelial migration (**Figure 5C**). Genes that were tolerized in both cell types in this pathway include *SELE*, *ICAM1* and *VCAM1*, which code for adhesion molecules and are induced by LPS in pericytes, a cell type that interacts with ECs (64). This finding suggests that the initial Poly(I:C) exposure induced genes necessary for the recruitment of phagocytes in the circulation, and that the subsequent tolerization of these genes is required to prevent excessive recruitment and inflammation. This is of course speculative and requires functional validation in co-culture experiments or *in vivo*.

It is important to note that culturing induces epigenetic and transcriptional changes in endothelial and other cell types, and that the media used for both cell types contains growth factors that partially activate the cells. As a result, ECFCs and HUVECs in culture may not represent the *in vivo* condition. Nevertheless, the different initial response to Poly(I:C) indicates that cell-intrinsic responses remain. Further, our model has a 24 hour rest period, which is similar to that used to study innate immune tolerance (5, 65), but not as long as that used to study trained immunity (66). As described in Koch et al. (17), there will still be some circulating cytokines in the supernatant after the 24 hour rest period, which would have a polarising effect on the cells. To address this, in our analysis we removed genes that show a persistent expression after Poly(I:C) and only designated genes as trained or attenuated if their basal expression at 48 hours was similar to that of media-exposed cells, in keeping with the definition of trained immunity and tolerance (4).

In summary, our data show that two distinct fetal endothelial cell types can both remodel their transcriptome in response to an inflammatory stimulus, with ECFCs inducing a distinct subset of genes from HUVECs in response to the viral ligand Poly(I:C), linked to the transcription factor ELF1. This remodelling in response to Poly(I:C) results in training or attenuation of specific gene sets in response to subsequent LPS stimulation, with a strong overlap between the two cell types. Genes that were tolerized in both cell types are involved in leukocyte recruitment, suggesting a mechanism that prevents excessive recruitment. Baseline genome-wide DNA methylation differences were a poor predictor of inflammatory transcriptional response, which is in line with previous work showing that acute inflammatory responses are regulated by histone modifications and nucleosome occupancy.

## Data Availability Statement

The data sets generated and analyzed for the current study are deposited in the Gene Expression Omnibus repository with the accession number GSE180881.

## Author Contributions

EW, AV, BK, DS, SW, and TA performed the cell culture experiments. J-HJ, RSh, JC, EW and UH provided primary endothelial cells. UH, MG, RS and BN supervised the students performing the experiments. RS and BN conceptualised the study. BN SW and SI performed the bioinformatic analysis. EW, UH and BN wrote the original draft manuscript. All authors contributed to the article and approved the submitted version.

## Funding

BN is supported by an NHMRC Investigator Grant (APP1173314) and an NHMRC Project Grant (APP1157556). UH and EW are supported through the PhD program Inflammatory Disorders in Pregnancy (DP-iDP) by the Austrian Science Fund FWF (Doc 31-B26) and the Medical University of Graz, Austria.

## Conflict of Interest

The authors declare that the research was conducted in the absence of any commercial or financial relationships that could be construed as a potential conflict of interest.

## Publisher’s Note

All claims expressed in this article are solely those of the authors and do not necessarily represent those of their affiliated organizations, or those of the publisher, the editors and the reviewers. Any product that may be evaluated in this article, or claim that may be made by its manufacturer, is not guaranteed or endorsed by the publisher.

## Supplementary Material

Supplementary Figure 1 Persistent Poly(I:C) induced gene expression. **(A)** Bar plot showing median expression of genes following Poly(I:C) exposure. Genes were separated into 4 quartiles based on expression difference at 48h between Poly(I:C) and media exposed cells (green line – ECFCs, black line – media). This time-point is 24h after removal of the stimulus. Q1 shows the most persistent expression, while genes in Q4 return to basal levels. **(B)** Expression of example genes in ECFCs and HUVECs exposed to Poly(I:C) or media. MX1 is the top gene showing persistent expression after Poly(I:C), CXCL10 is in Q2, and is showing reduced expression at 48h, while TNF is in Q4 and shows complete return to basal levels by 48h. **(C)** Bar plot showing abundance of TF motifs at the 4 quartiles. Q1 is enriched for IRF, NFKB and STAT motifs, with no clear enrichment of motifs at the other quartiles.

Supplementary Figure 2 Poly(I:C) associated transcriptional programs are dependent on direct exposure. **(A)** Experimental model. **(B)** Gene expression after indirect Poly(I:C) exposure, i.e. cells were seeded after incubating wells with stimulation media following wash out, is comparable to media control.

Supplementary Figure 3 Motif enrichment scores and expression of associated transcription factors involved in the initial Poly(I:C) response in ECFCs and HUVECs. **(A)** Abundance of transcription factor motifs in promotors of Poly(I:C) induced genes. **(B)** Transcription factor genes related to the motif signature are higher in HUVECs than ECFCs.

Supplementary Figure 4 Summary of genes trained or tolerized for LPS response by Poly(I:C) in HUVECs and ECFCs. **(A)** PCA plot of transcriptomic changes that occur 4 hours following LPS exposure after previous stimulation with Poly(I:C) or media only. **(B)** Expression of trained, unaffected and tolerized genes over time in HUVECs. Genes trained for LPS response are more strongly induced by Poly(I:C) compared to genes that are tolerized for LPS response. **(C)** Line plot showing expression of example trained, unaffected and tolerized genes (green line – ECFCs, blue line – HUVECs, black line – media control).
