# Supplementary figures and images for: Transcriptomic Remodelling of Fetal Endothelial Cells During Establishment of Inflammatory Memory
Source: Front Immunol. 2021 Nov 19;12:757393. doi: 10.3389/fimmu.2021.757393 (PMC8640490; doi:10.3389/fimmu.2021.757393)

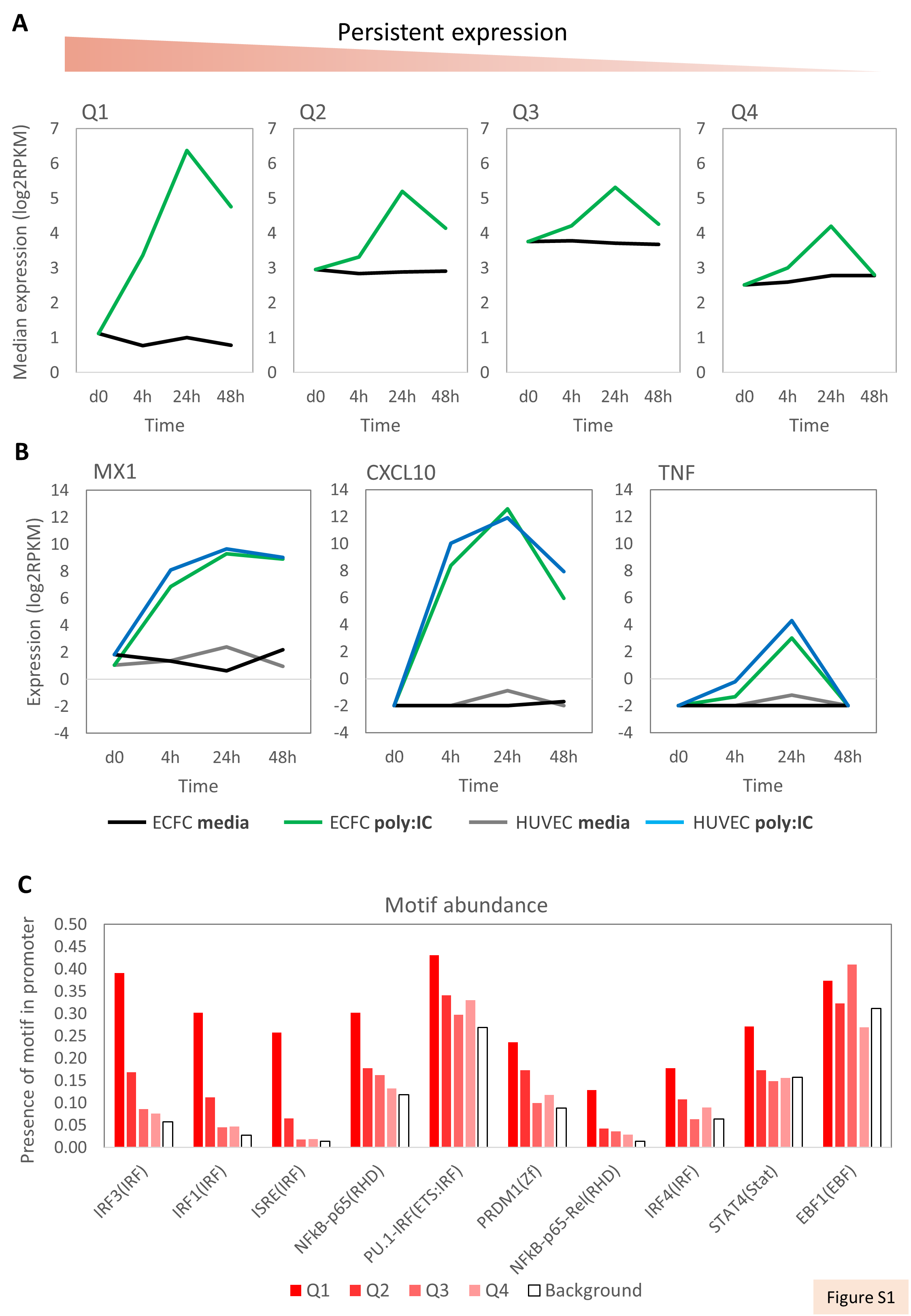

Supplement: Supplementary Figure 1 — Persistent Poly(I:C) induced gene expression. (A) Bar plot showing median expression of genes following Poly(I:C) exposure. Genes were separated into 4 quartiles based on expression difference at 48h between Poly(I:C) and media exposed cells (green line – ECFCs, black line – media). This time-point is 24h after removal of the stimulus. Q1 shows the most persistent expression, while genes in Q4 return to basal levels. (B) Expression of example genes in ECFCs and HUVECs exposed to Poly(I:C) or media. MX1 is the top gene showing persistent expression after Poly(I:C), CXCL10 is in Q2, and is showing reduced expression at 48h, while TNF is in Q4 and shows complete return to basal levels by 48h. (C) Bar plot showing abundance of TF motifs at the 4 quartiles. Q1 is enriched for IRF, NFKB and STAT motifs, with no clear enrichment of motifs at the other quartiles. [file Image_1.tif]

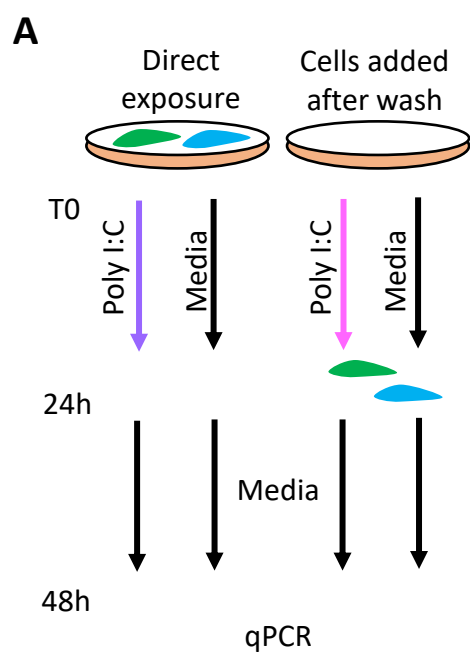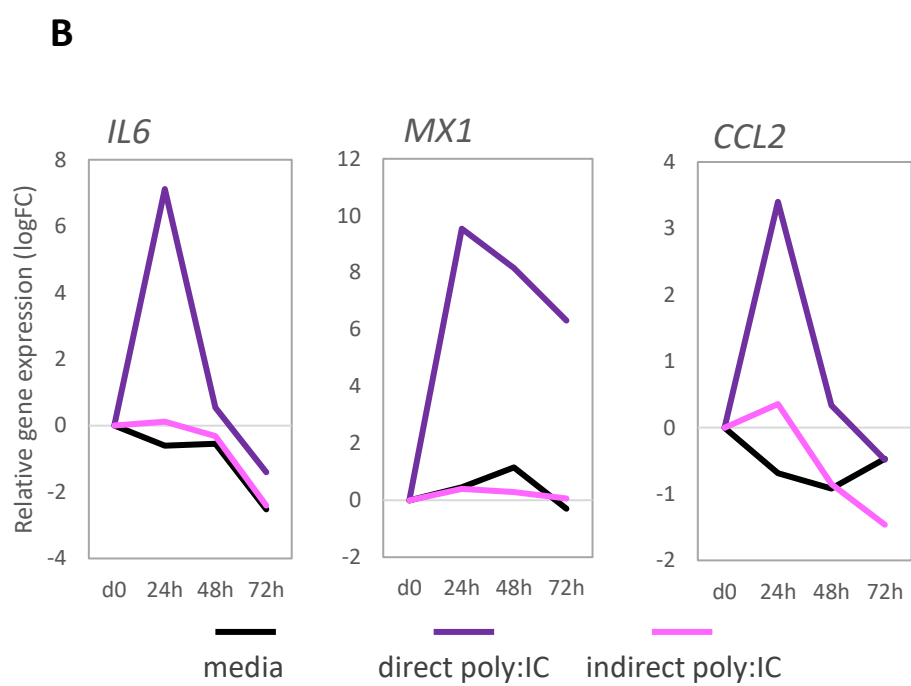

Figure S2

Supplement: Supplementary Figure 2 — Poly(I:C) associated transcriptional programs are dependent on direct exposure. (A) Experimental model. (B) Gene expression after indirect Poly(I:C) exposure, i.e. cells were seeded after incubating wells with stimulation media following wash out, is comparable to media control. [file Image_2.pdf]

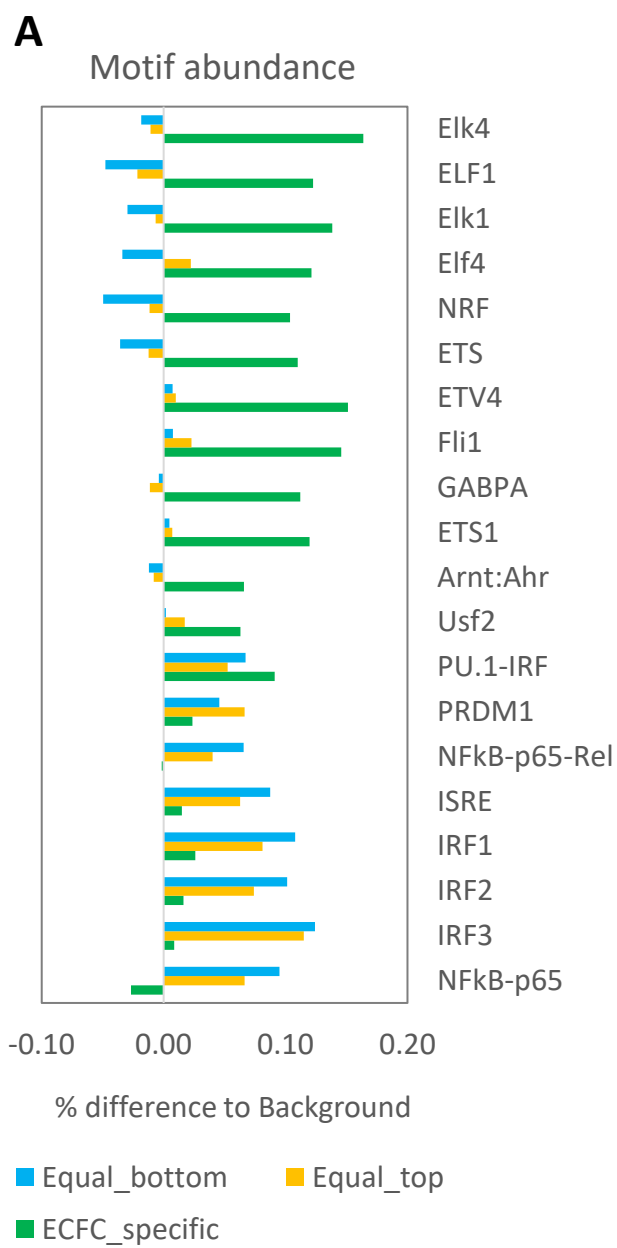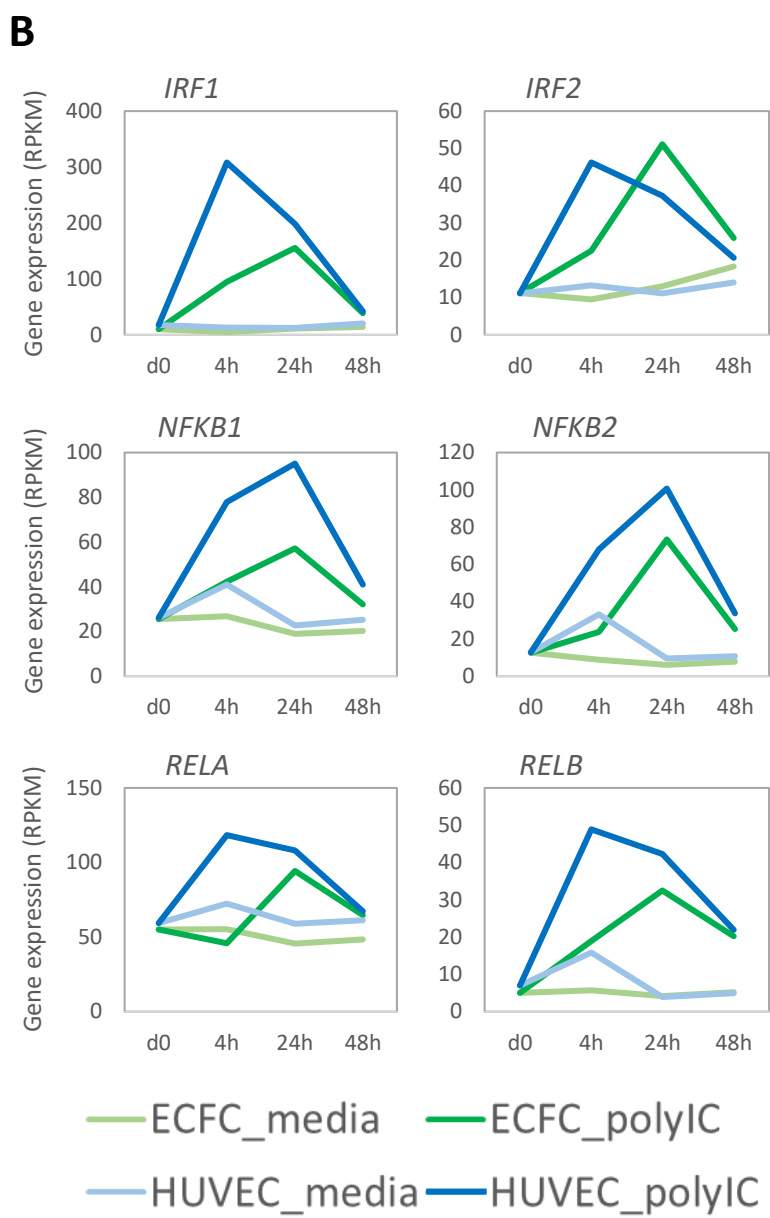

Figure S3

Supplement: Supplementary Figure 3 — Motif enrichment scores and expression of associated transcription factors involved in the initial Poly(I:C) response in ECFCs and HUVECs. (A) Abundance of transcription factor motifs in promotors of Poly(I:C) induced genes. (B) Transcription factor genes related to the motif signature are higher in HUVECs than ECFCs. [file Image_3.pdf]

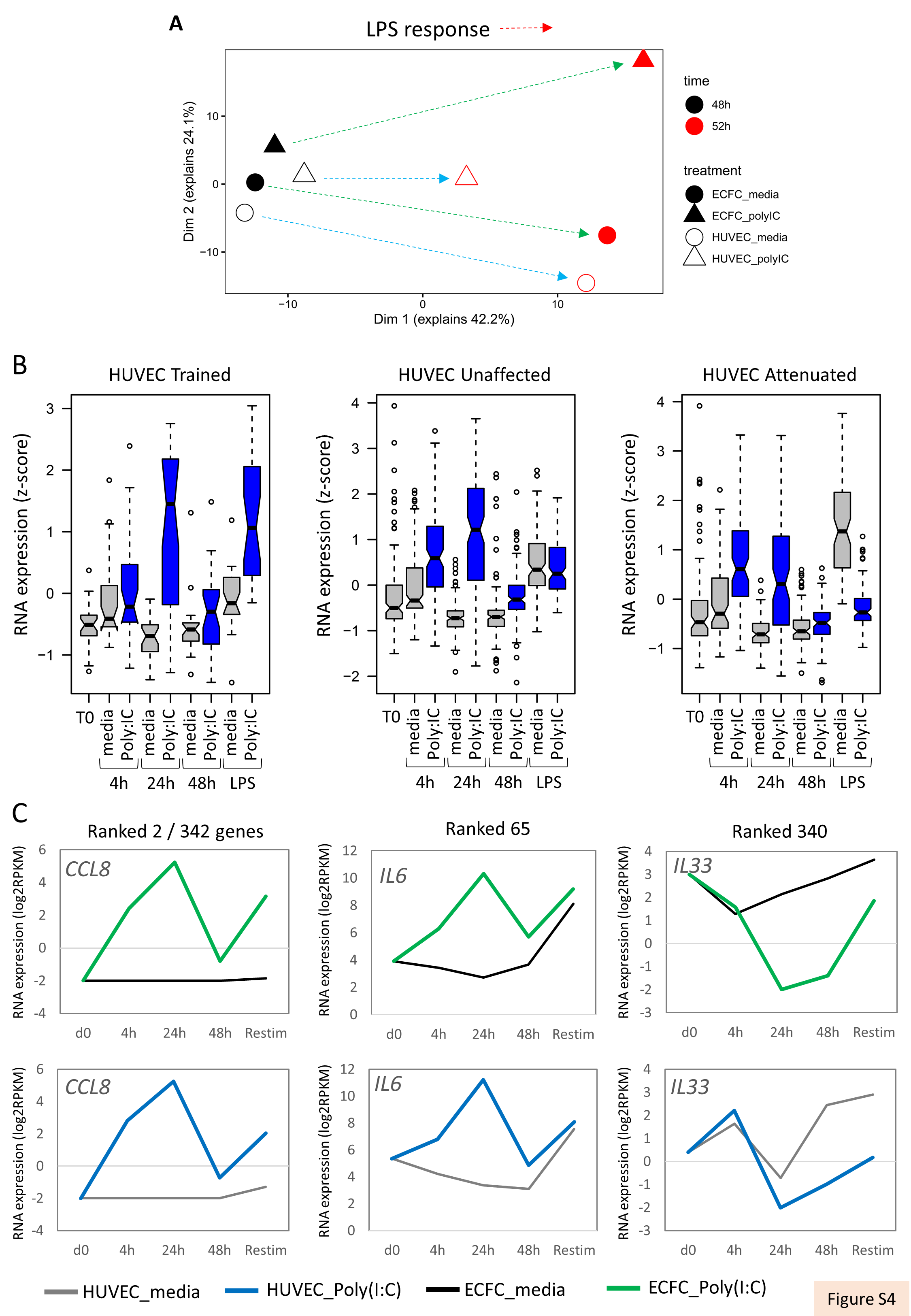

Supplement: Supplementary Figure 4 — Summary of genes trained or tolerized for LPS response by Poly(I:C) in HUVECs and ECFCs. (A) PCA plot of transcriptomic changes that occur 4 hours following LPS exposure after previous stimulation with Poly(I:C) or media only. (B) Expression of trained, unaffected and tolerized genes over time in HUVECs. Genes trained for LPS response are more strongly induced by Poly(I:C) compared to genes that are tolerized for LPS response. (C) Line plot showing expression of example trained, unaffected and tolerized genes (green line – ECFCs, blue line – HUVECs, black line – media control). [file Image_4.tif]
